# Investigation of vitamin D deficiency in girls with growth and development variations—a single center study

**DOI:** 10.3389/fped.2025.1518548

**Published:** 2025-01-27

**Authors:** Panwang Huang, Beilei Zeng, Feng Ren, Yuan Zhou, Ye Li, Yinyin Huang, Xingyu Liu, Jiaxiu Zhou, Yaping Ma

**Affiliations:** ^1^ Department of Pediatrics, Affiliated Hospital of Jiangnan University, Wuxi, Jiangsu, China; ^2^ Laboratory of Genomic and Precision Medicine, Wuxi School of Medicine, Jiangnan University, Wuxi, Jiangsu, China; ^3^ Department of Clinical Laboratory, Affiliated Hospital of Jiangnan University, Wuxi, Jiangsu, China

**Keywords:** growth and developmental variations, girls, vitamin D deficiency, early puberty, menarche

## Abstract

**Purpose:**

To understand the status of vitamin D deficiency in girls with growth and developmental variations, as well as the impact of COVID−19 on their vitamin D levels, and to provide reference for the prevention and treatment of vitamin D deficiency in children.

**Methods:**

A retrospective analysis was conducted on 1,345 instances of girls with growth and developmental variations who visited our pediatric endocrinology department and completed vitamin D detection. A total of 279 girls with complete data were included in this study. Among them, 246 girls were classified into four groups based on different growth and developmental variations: early puberty group, menarche group, obesity group, short stature group, and 33 healthy girls served as the control group. Besides, the girls were divided into pre-epidemic and post-epidemic groups by the occurrence of the COVID-19 epidemic. Vitamin D were measured in all girls. The 25(OH)D <20 ng/ml was used as the standard for vitamin D deficiency.

**Results:**

The levels of vitamin D in the early puberty group, menarche group, obesity group, short stature group, and control group were as follows: 20.23 ± 5.90 ng/ml, 17.85 ± 5.69 ng/ml, 21.31 ± 8.99 ng/ml, 27.90 ± 12.27 ng/ml, and 29.42 ± 12.65 ng/ml, respectively. The levels of vitamin D in the early puberty group, menarche group, and obesity group were significantly lower than those in the control group (*P* < 0.05). The individual vitamin D deficiency rates in the aforementioned groups were 52.07%, 73.91%, 59.46%, 30.95%, and 30.30%, respectively. The vitamin D levels in the pre-epidemic and post-epidemic groups were 20.48 ± 6.22 ng/ml and 22.50 ± 9.74 ng/ml, respectively (*P* > 0.05).

**Conclusion:**

Girls with early puberty, menarche, and obesity have a certain deficiency of vitamin D levels, and appropriate vitamin D treatment should be provided clinically. Girls with short stature and healthy girls also have certain levels of vitamin D deficiency, and awareness of prevention should be strengthened.

## 1 Introduction

Vitamin D, a fat-soluble vitamin derived from steroids, is essential for maintaining the life of higher animals and plays a vital role in individual development (1). Especially in the growth and development of children, vitamin D deficiency can lead to various diseases such as rickets and osteoporosis (2). Therefore, timely prevention and supplementation of vitamin D are particularly important for children with growth and developmental variations. In addition, vitamin D is also associated with various pubertal development disorders in children. It was shown that Vitamin D levels decreased in children with precocious puberty, obesity, and polycystic ovary syndrome (3–5). However, the specific mechanisms of its effects remain to be further studied.

The aim of this study is to analyze the deficiency of vitamin D in girls with different growth and developmental variations. Moreover, it may provide some clinical reference data for preventive and timely vitamin D supplementation in girls.

## 2 Subjects and methods

### 2.1 Study subjects

A retrospective analysis was performed on 1,345 instances of girls who visited the pediatric Eendocrinology department of affiliated hospital of Jiangnan University and completed vitamin D detection between March 16, 2,018, and October 17, 2023. To eliminate factors such as previous treatment or medical history, only girls with growth and development abnormalities at their first visit were screened for the study. Ultimately, 279 girls with an average age of 9.2 years were enrolled, including 121 cases of early puberty, 46 cases of menarche, 37 cases of obesity, 42 cases of short stature, and 33 healthy girls. This study was approved by the Ethics Committee in our hospital.

### 2.2 Detection methods

Vitamin D was measured using the electrochemiluminescence immunoassay analyzer (Cobas 8000e602). Luteinizing hormone (LH), follicle-stimulating hormone (FSH), and estrogen (E2) were detected by ACCESS Automatic Microparticle Immunochemiluminescence Analyzer (DxI800, BECKMAN, USA). Parathyroid hormone (PTH) was performed on an automated assay (LIASON® N-TACT®, DiaSorin). Alkaline phosphatase (ALP) was detected by Alkaline phosphatase test reagent kit (Method of NPP-AMP).

### 2.3 Growth and development assessment

The growth and development growth parameters were included. Height and weight were measured by standardized methods (6). According to Tanner stage (7), pubertal development in girls is divided into 5 stages related to changes that are observed in the development of breasts and pubic hair. x-ray examination of the left wrist joint was performed, and bone age (BA) was calculated by TW2R method. Clinical indicators such as bone age and Tanner stage were assessed by two endocrinology experts.

### 2.4 Diagnosis criteria

Girls with precocious puberty and early puberty were all included in the early puberty group. The diagnosis criteria for precocious puberty and early puberty in girls refer to the expert consensus on diagnosis and treatment of central precocious puberty (8). Precocious puberty is defined as the development of secondary sexual characteristics in girls before 8 years of age or the onset of menarche before 10 years of age. Pelvic ultrasound indicates enlargement of the uterus and ovaries. Serum gonadotropins (Gn) and sex hormones reach the level of puberty. Bone age advances by more than 1 year. Linear growth is accelerated. Early puberty refers to the appearance of secondary sexual characteristics in girls after 8 years of age and before the average age of normal puberty (9).

The inclusion criteria for the menarche group required participants to be girls experiencing their first menstrual period, who consequently sought consultation in the pediatric endocrinology department for assessment of their developmental status.

For the obesity group, the diagnosis criteria are based on the recommendations of the Working Group on Obesity in China. Obesity is defined as a body mass index (BMI) exceeding the 95th percentile for the same gender and age, excluding other endocrine disorders causing obesity (10).

The diagnosis criteria for the short stature group are based on the guidelines for the diagnosis and treatment of idiopathic short stature (ISS) in children (11). ISS refers to a clinically heterogeneous group of growth disorders characterized by unexplained short stature. It is defined as a height that is more than 2 standard deviations (SD) below the mean or below the 3rd percentile (P3, −1.88 SD) for individuals of the same age, sex, and ethnic background. Individuals with ISS typically exhibit normal birth length, birth weight, and body proportions, with no detectable evidence of systemic disease, endocrine dysfunction, nutritional deficiency, chromosomal abnormalities, or genetic mutations (12).

The grouping criteria before and after the epidemic are defined based on the outbreak of the epidemic. All 279 girls in this study were Girls divided by who visited the hospital before December 19, 2019, are defined as the pre-epidemic group (*n* = 39), while those after December 19, 2019, are defined as the post-epidemic group (*n* = 240).

According to the diagnostic criteria for vitamin D deficiency (5), vitamin D level <10 ng/ml (25 nmol/L) is defined as vitamin D severe deficiency, <20 ng/ml (50 nmol/L) as vitamin D deficiency, between 20 and 30 ng/ml as vitamin D insufficiency, and >30 ng/ml as vitamin D sufficiency (13).

The exclusion criteria encompassed girls diagnosed with liver diseases, kidney diseases, thyroid disorders, parathyroid dysfunction, adrenal disorders, bone metabolism abnormalities, or other conditions affecting calcium homeostasis or hypothalamic-pituitar*y* axis function. Girls undergoing corticosteroid treatment, as well as those with chromosomal abnormalities, internal organ malformations, malabsorption disorders, or congenital metabolic diseases, were also excluded from the study.

### 2.5 Statistical analysis

Data analysis was performed using SPSS 25.0 statistical software. Graphs were plotted using Graphpad Prism 9.5.0 software. Measurement data were presented as mean ± standard deviation (X ± SD). Non-parametric tests were used for comparisons between the early puberty group, menarche group, obesity group, and short stature group with the control group, as well as for comparisons between pre-epidemic and post-epidemic groups. The calculation of Standardized Mean Differences (SMD) for basic clinical indicators and the creation of forest plots were performed using R (version 4.3.1). Correlation heatmaps were also generated using R. The linear regression analysis for the comparison of indicators and vitamin D levels across different groups was conducted using SPSS. Spearman correlation analysis was used to assess the correlation between two independent variables. *P* < 0.05 was considered the difference to be statistically significant.

## 3 Results

### 3.1 Basic characteristics of girls

**Figure 1:**
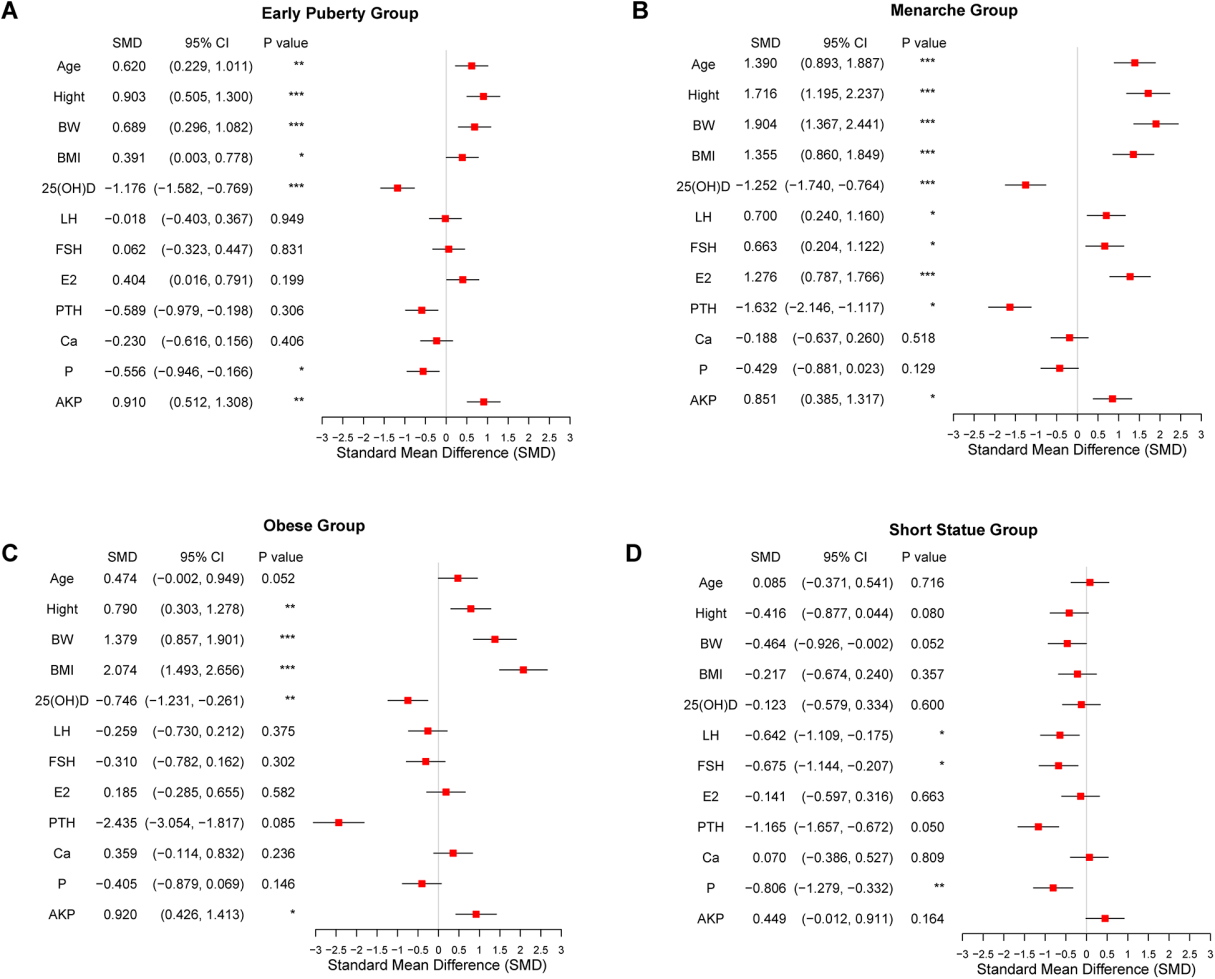
Forest plots comparing the clinical physiological indicators between developmental variations groups and the control group. **(A)** Early puberty group. **(B)** Menarche group. **(C)** Obesity group. **(D)** Short stature group. Significant differences between groups are indicated by *P < 0.05, **P < 0.01, or ***P < 0.001.

A total of 279 pubertal girls were assigned to this study, including 246 girls with growth and developmental variations and 33 girls with normal development. Based on the different diagnoses of growth and development abnormalities, the 246 girls were divided into 4 groups: the early puberty group, menarche group, obesity group, and short stature group. 33 girls with normal development were enrolled in the control group. The basic clinical characteristic of the 279 girls is shown in Table 1. The estrogen levels in the menarche group were significantly higher than those in the control group (*P* < 0.001). The BMI of the obesity group and menarche group was significantly higher than that of the control group (*P* < 0.001). The LH levels in the menarche groups was significantly elevated compared to the control group (*P* < 0.05). We also measured alkaline phosphatase levels among the groups, and the results showed a strong correlation between the groups with elevated alkaline phosphatase levels and those groups with decreased vitamin D levels. Moreover, the difference was significant. To provide a more intuitive understanding of the comparison of physiological indicators between girls in the four abnormal development groups and the control group, we also generated forest plots for the Standardized Mean Differences (SMD) (Figures 1A–D). These plots illustrate the SMD for various physiological markers across the four development variations groups in comparison to the control group.

**Table 1:** Basic clinical characteristic of girls with growth and development variations.

	Early puberty group (*n* = 121)	Menarche group (*n* = 46)	Obesity group (*n* = 37)	Short stature group (*n* = 42)	Control group (*n* = 33)
Age (y)	9.21 ± 1.44^a^	10.91 ± 1.14^a^	9.25 ± 2.04^a^	8.32 ± 2.82	8.08 ± 2.86
Height (cm)	136.66 ± 8.94^a^	148.38 ± 7.85^a^	138.38 ± 13.13^a^	119.68 ± 15.34	126.46 ± 17.49
Weight (kg)	31.53 ± 7.80^a^	43.69 ± 9.26^a^	43.24 ± 14.80^a^	22.14 ± 6.90	25.90 ± 9.46
BMI	16.70 ± 2.41	19.62 ± 3.12^a^	21.86 ± 3.33^a^	15.18 ± 2.80	15.75 ± 2.42
Bone age (y)	10.21 ± 1.4^a^	12.02 ± 0.78^a^	9.70 ± 1.68^a^	7.75 ± 2.20	7.96 ± 1.55
Tanner stage	2.51 ± 0.94^a^	3.89 ± 0.98^a^	2.61 ± 1.23^a^	1.53 ± 0.76	1.77 ± 1.18
LH (IU/L)	2.03 ± 4.93	5.96 ± 6.13^a^	1.25 ± 2.15	0.26 ± 0.41	2.13 ± 4.37
FSH (IU/L)	4.65 ± 4.19	6.18 ± 2.21	3.59 ± 1.91	2.71 ± 1.75	4.41 ± 3.32
E2 (pg/ml)	32.90 ± 37.70^a^	47.15 ± 27.47^a^	21.02 ± 9.59	18.61 ± 8.10	19.27 ± 9.31
PTH (ng/L)	33.77 ± 22.23	25.42 ± 10.42	19.65 ± 3.89^a^	30.39 ± 11.91	46.11 ± 15.29
Ca (mmol/L)	2.47 ± 0.10	2.48 ± 0.08	2.53 ± 0.09	2.50 ± 0.10	2.49 ± 0.10
*P* (mmol/L)	1.68 ± 0.17	1.70 ± 0.17	1.70 ± 0.14	1.62 ± 0.15^a^	1.79 ± 0.27
ALP (U/L)	298.17 ± 92.51^a^	275.16 ± 66.14^a^	286.45 ± 79.26^a^	243.33 ± 44.24	218.18 ± 68.09

BMI, body mass index, Bone age, years; LH, luteinizing hormone (IU/L); FSH, follicle-stimulating hormone (IU/L); E2, estradiol (pg/ml); PTH, parathyroid hormone (ng/L); Ca, calcium (mmol/L); P, phosphorus (mmol/L); ALP, alkaline phosphatase (U/L).

^a^ Significant difference compared to the control group.

### 3.2 Differences of vitamin D levels in girls with growth and development variations

**Figure 2:**
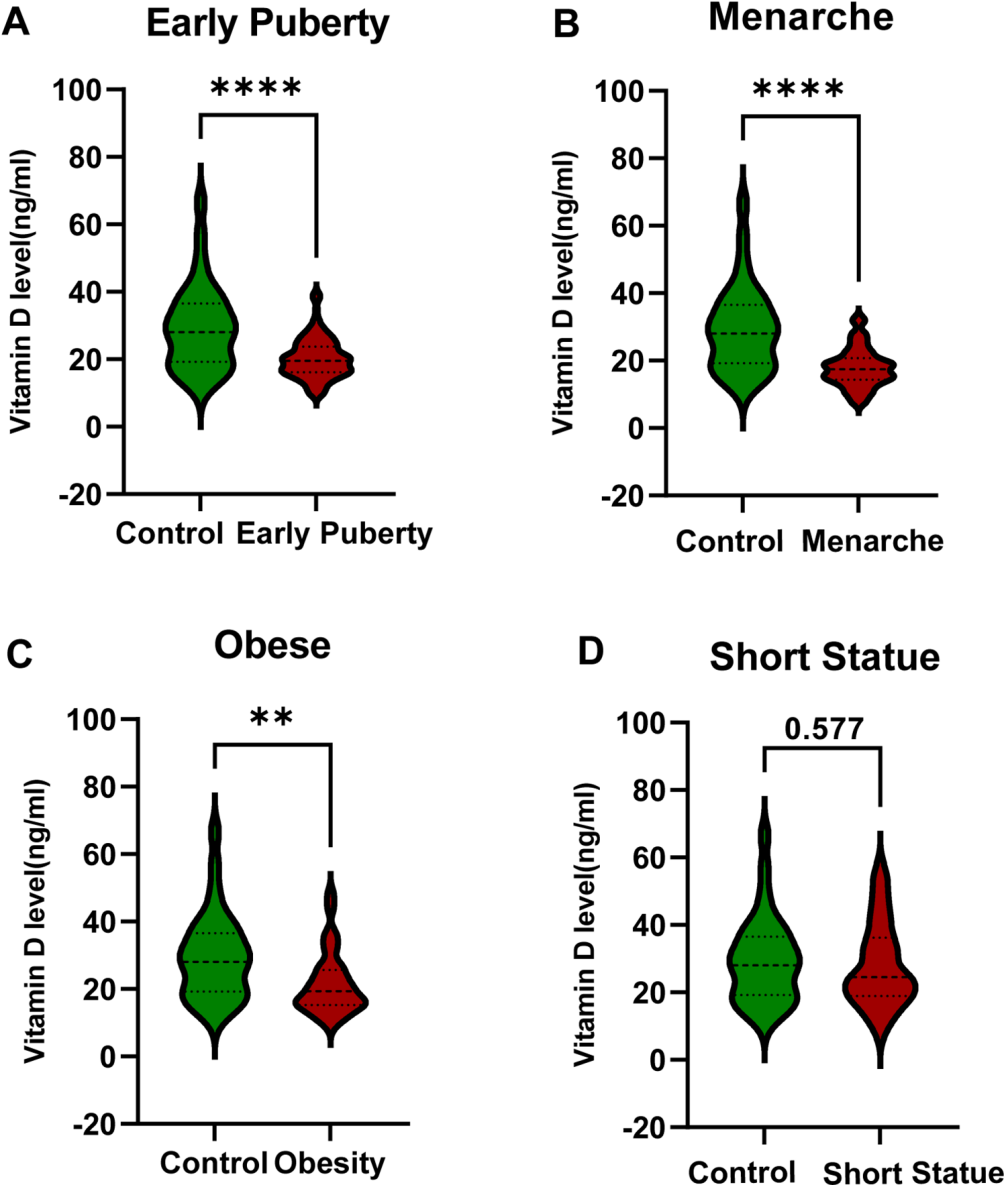
Comparison of vitamin D levels between developmental variations groups and the control group. **(A)** Early Puberty Group. **(B)** Menarche Group. **(C)** Obesity Group. **(D)** Short Stature Group. Statistical comparisons were conducted using non-parametric test. Significant differences between groups are indicated by *P < 0.05, **P < 0.01, or ***P < 0.001.

The average vitamin D levels in the early puberty group, menarche group, obesity group, short stature group, and control group were 20.23 ± 5.90 ng/ml, 17.85 ± 5.69 ng/ml, 21.31 ± 8.99 ng/ml, 27.90 ± 12.27 ng/ml, and 29.42 ± 12.65 ng/ml, respectively. The comparison of vitamin D levels among different groups revealed that the vitamin D levels in the early puberty, menarche, and obesity groups were significantly lower than the control group, with statistical significance (Figures 2A–C). The difference in vitamin D levels between the short stature group and the control group was not statistically significant (*P* > 0.05) (Figure 2D). The ALP levels exhibited a high degree of consistency with vitamin D levels across the groups (Supplementary Figure 1A).

### 3.3 Linear regression analysis of vitamin D levels and various physiological indicators

Based on the results 2, it can be observed that the vitamin D levels in girls from the early puberty, menarche, and obesity groups are significantly lower than those in the control group. However, there are differences in age between these groups, which could influence the results. To account for this, we conducted a linear regression analysis of vitamin D levels and various physiological indicators within each group to control for the influence of other factors on vitamin D.

The results indicate that, in the early puberty group, the regression coefficient for age is −0.66, with a 95% confidence interval of (−1.40, 0.07) and a *p*-value of 0.07. This suggests a potential negative association between age and vitamin D levels; however, this relationship is not statistically significant at the conventional 0.05 level. Similarly, in the menarche group, the *p*-value for age is also greater than 0.05, indicating that age does not significantly impact vitamin D levels in these two groups. While there is a trend suggesting lower vitamin D levels with increasing age, the effect is not strong enough to be deemed significant in this analysis. A significant negative effect on vitamin D levels is observed with a regression coefficient of −2.21 (*P* = 0.01), suggesting that higher BMI is associated with lower vitamin D levels in early puberty group (Table 2).

**Table 2:** Liner regression analysis of vitamin D levels and various physiological indicators.

	Early puberty group			Menarche group			Obesity group			Short stature group			Control group		
	B	95% CI	*P* value	B	95% CI	*P* value	B	95% CI	*P* value	B	95% CI	*P* value	B	95% CI	*P* value
Age (y)	−0.66	(−1.4, 0.07)	0.07	0.68	(−0.83, 2.18)	0.37	−2.42^b^	(−3.68, −1.16)	0.01	−2.12^b^	(−3.33, −0.9)	0.01	−1.73^b^	(−3.23, −0.24)	0.02
Height (cm)	−0.19^b^	(−0.3, −0.07)	0.01	0.11	(−0.11, 0.33)	0.32	−0.33^b^	(−0.54, −0.13)	0.01	−0.32^b^	(−0.56, −0.09)	0.01	−0.33^b^	(−0.57, −0.1)	0.01
Weight (kg)	−0.2^b^	(−0.33, −0.07)	0.01	−0.09	(−0.28, 0.1)	0.35	−0.3^b^	(−0.48, −0.12)	0.01	−0.75^b^	(−1.27, −0.23)	0.01	−0.55^b^	(−1, −0.11)	0.02
BMI	−0.39	(−0.83, 0.05)	0.08	−0.59	(−1.15, −0.03)	0.04	−1.26^b^	(−2.08, −0.45)	0.01	−0.76	(−2.15, 0.64)	0.28	−0.8	(−2.69, 1.09)	0.39
Bone age (y)	−2.21^b^	(−3.48, −0.94)	0.01	−2.18^b^	(−3.98, −0.38)	0.02	−1.25	(−4.18, 1.68)	0.39	−2.41	(−7.91, 3.09)	0.38	−2.27	(−5.79, 1.25)	0.2
Tanner stage	−0.76^b^	(−1.51, −0.02)	0.04	−1.01	(−3.62, 1.59)	0.43	−1.16	(−3.58, 1.25)	0.33	−2^b^	(−3.74, −0.27)	0.03	0.36	(−2.69, 3.41)	0.8
LH (IU/L)	−0.18^b^	(−0.41, 0.06)	0.14	0.03	(−0.27, 0.33)	0.82	−0.7	(−2.29, 0.9)	0.38	0.15	(−10.53, 10.83)	0.98	−1.25	(−2.51, 0.01)	0.05
FSH (IU/L)	−0.09	(−0.38, 0.19)	0.52	−0.15	(−0.99, 0.68)	0.71	−0.45	(−2.26, 1.36)	0.61	1.19	(−1.24, 3.62)	0.32	−1.14	(−3.02, 0.73)	0.21
E2 (pg/ml)	0	(−0.04, 0.03)	0.84	0	(−0.06, 0.07)	0.9	0.05	(−0.32, 0.41)	0.79	−0.35	(−0.95, 0.26)	0.25	−0.21	(−0.94, 0.52)	0.54
PTH (ng/L)	−0.1	(−0.2, −0.01)	0.04	−0.34	(−0.92, 0.24)	0.19	−0.54^a^	(−0.54, −0.54)	0.001	−0.2	(−0.95, 0.55)	0.56	−0.94	(−2.17, 0.3)	0.08
Ca (mmol/L)	16.13^b^	(1.42, 30.83)	0.03	−13.17	(−37.2, 10.86)	0.27	−9.48	(−45.06, 26.09)	0.59	53.48^b^	(16.91, 90.06)	0.01	73.47^b^	(8.65, 138.29)	0.03
*P* (mmol/L)	−1.15	(−10.55, 8.24)	0.81	−3.27	(−14.73, 8.18)	0.56	12.24	(−13, 37.49)	0.33	7.99	(−20.08, 36.06)	0.57	−4.92	(−31.6, 21.77)	0.7
ALP (U/L)	0	(−0.02, 0.01)	0.62	−0.01	(−0.04, 0.02)	0.43	0	(−0.05, 0.05)	0.97	0	(−0.09, 0.1)	0.96	0.03	(−0.12, 0.19)	0.63

^a^ BMI, body mass index; Bone age, years; LH, luteinizing hormone (IU/L); FSH, follicle-stimulating hormone (IU/L); E2, estradiol (pg/ml); PTH, parathyroid hormone (ng/L); Ca, calcium (mmol/L); P, phosphorus (mmol/L); ALP, alkaline phosphatase (U/L).

^b^ Significant difference compared to the control group.

### 3.4 Comparison of vitamin D deficiency rates among groups

To explore the proportions of vitamin D deficiency among different groups and the severity of deficiency within each group, we further analyzed the vitamin D deficiency rates among the groups. The individual vitamin D deficiency rates in the early puberty group, menarche group, obesity group, short stature group, and control group were 52.07%, 73.91%, 59.46%, 30.95%, and 30.30%, respectively. The individual vitamin D insufficiency rates were 93.04%, 95.65%, 83.78%, 61.90%, and 57.58%, respectively. Among the groups of girls with growth and developmental variations, the menarche group had the highest individual deficiency rate, while the short stature group had the lowest. It is noteworthy that the combined rate of deficiency in the control group has even reached 30.30% (Table 3).

**Table 3:** Comparison of vitamin D deficiency rates in girls with growth and development variations.

Vitamin D Deficiency Rate		Early Puberty group (*n* = 121)	Menarche group (*n* = 46)	Obesity group (*n* = 37)	Short Stature group (*n* = 42)	Control group (*n* = 33)	Total (*n* = 279)
Severe deficiency	Count	3^a^	3^a^	1^a^	1^a^	0^a^	8
	Percentage	37.5%	37.5%	12.5%	12.5%	0.0%	100.0%
Deficiency	Count	60^a^	31^b^	21^a, b^	12^c^	10^c^	134
	Percentage	44.8%	23.1%	15.7%	9.0%	7.5%	100.0%
Insufficiency	Count	52^a^	10^b^	9^b^	13^a, b^	9^a, b^	93
	Percentage	55.9%	10.8%	9.7%	14.0%	9.7%	100.0%
Sufficiency	Count	6^a^	2^a, b^	6^b^	16^c^	14^c^	44
	Percentage	13.6%	4.5%	13.6%	36.4%	31.8%	100.0%
All deficiency	Percentage	52.07%	73.91%	59.46%	30.95%	30.30%	50.90%

Each subscript letter (a–c) represents a subset of categories within the diagnostic group. At the *P* = 0.05 level, these letters indicate statistically significant differences between the subsets.

### 3.5 Correlation analysis between vitamin D and basic clinical characteristics

**Figure 3:**
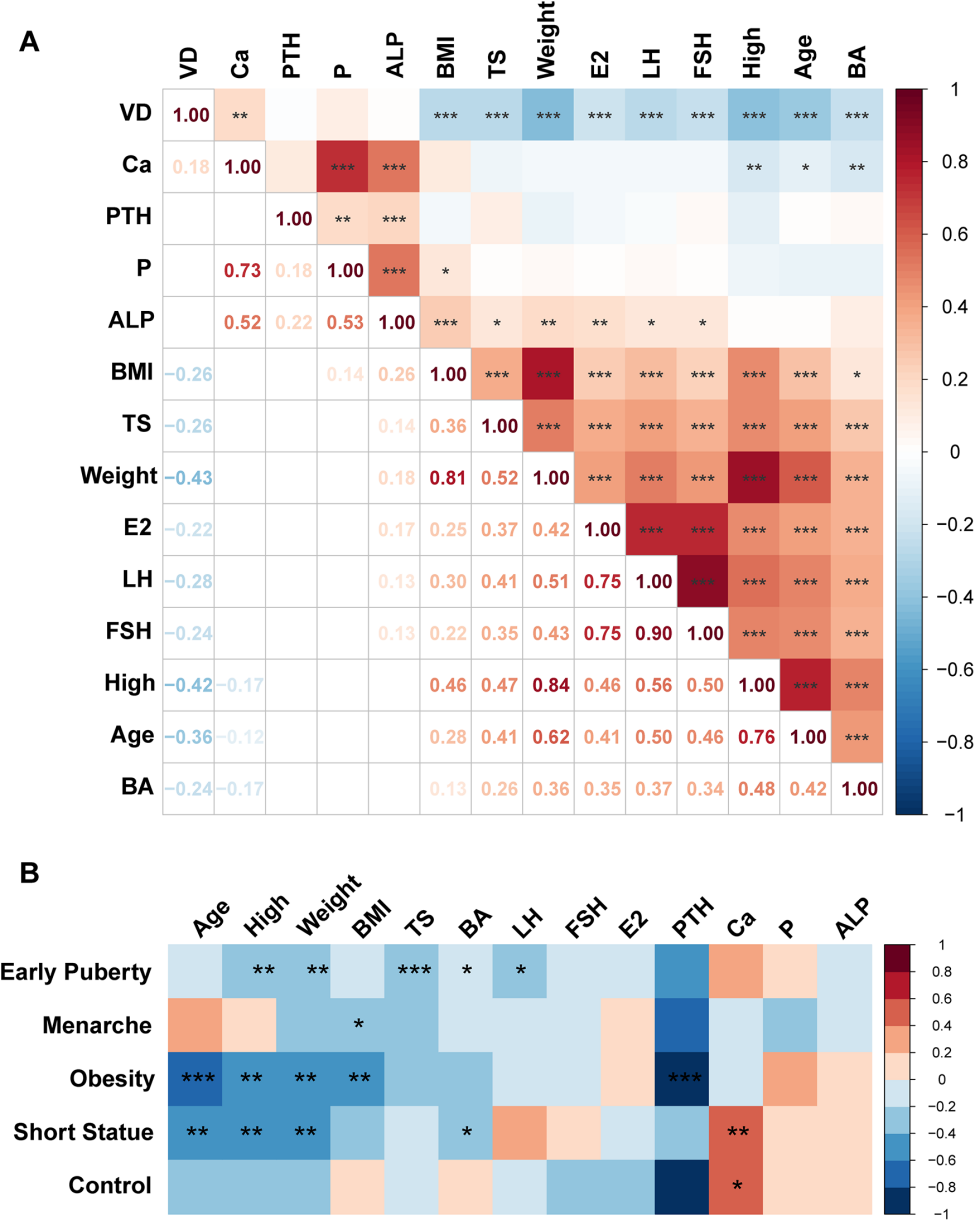
Correlation analysis of physiological indicators in girls. **(A)** Correlation of basic characteristic indicators in 279 girls in the overall group. **(B)** Correlation between vitamin D levels and other basic characteristic indicators within each group. Significant are indicated by *P < 0.05, **P < 0.01, or ***P < 0.001.

To investigate the relationship between vitamin D levels and other physiological indicators, we conducted a correlation analysis on 279 girls included in this study. The results revealed a significant positive correlation between vitamin D levels and blood calcium (R = 0.18) (*P* < 0.05). In contrast, vitamin D levels were negatively correlated with body weight, height, age, BMI, and Tanner stage (R = −0.43, R = −0.42, R = −0.36, R = −0.26, R = −0.26) (*P* < 0.01). Additionally, significant negative correlations were observed between vitamin D levels and the hormonal indicators of E2, LH, and FSH (R = −0.22, R = −0.28, R = −0.24) (*P* < 0.01) (Figure 3A). Correlation analysis within each group showed a negative correlation between vitamin D levels and Tanner stage, Bone age, LH in the early puberty group (R = −0.333, R = −0.191, R = −0.236) (*P* < 0.05) (Figure 3B). In the menarche group, a negative correlation was found between vitamin D levels and BMI in (R = −0.326) (*P* < 0.05). Additionally, a significant negative correlation between vitamin D levels and BMI was observed in the obesity group (R = −0.467, *P* < 0.001). Besides, there was a negative correlation between vitamin D levels and bone age and a positive correlation between vitamin D levels and blood calcium in the short stature group (R = −0.387, R = 0.500) (*P* < 0.05, *P* < 0.01). Interestingly, we found that in the control group, there was a significant positive correlation between vitamin D levels and Ca levels (R = 0.506, *P* < 0.05).

### 3.6 Investigation of vitamin D differences before and after the epidemic

To investigate the impact of the COVID-19 pandemic on vitamin D levels in children, we divided the 279 female participants into pre-pandemic and post-pandemic groups. The vitamin D levels were 20.48 ± 6.22 ng/ml in the pre-epidemic group and 22.50 ± 9.74 ng/ml in the post-epidemic group, with no significant difference between these two groups. Therefore, the epidemic may not have a direct significant effect on the vitamin D levels of girls (Supplementary Figure 1B).

### 3.7 The follow-up for the status of vitamin D supplementation

**Figure 4:**
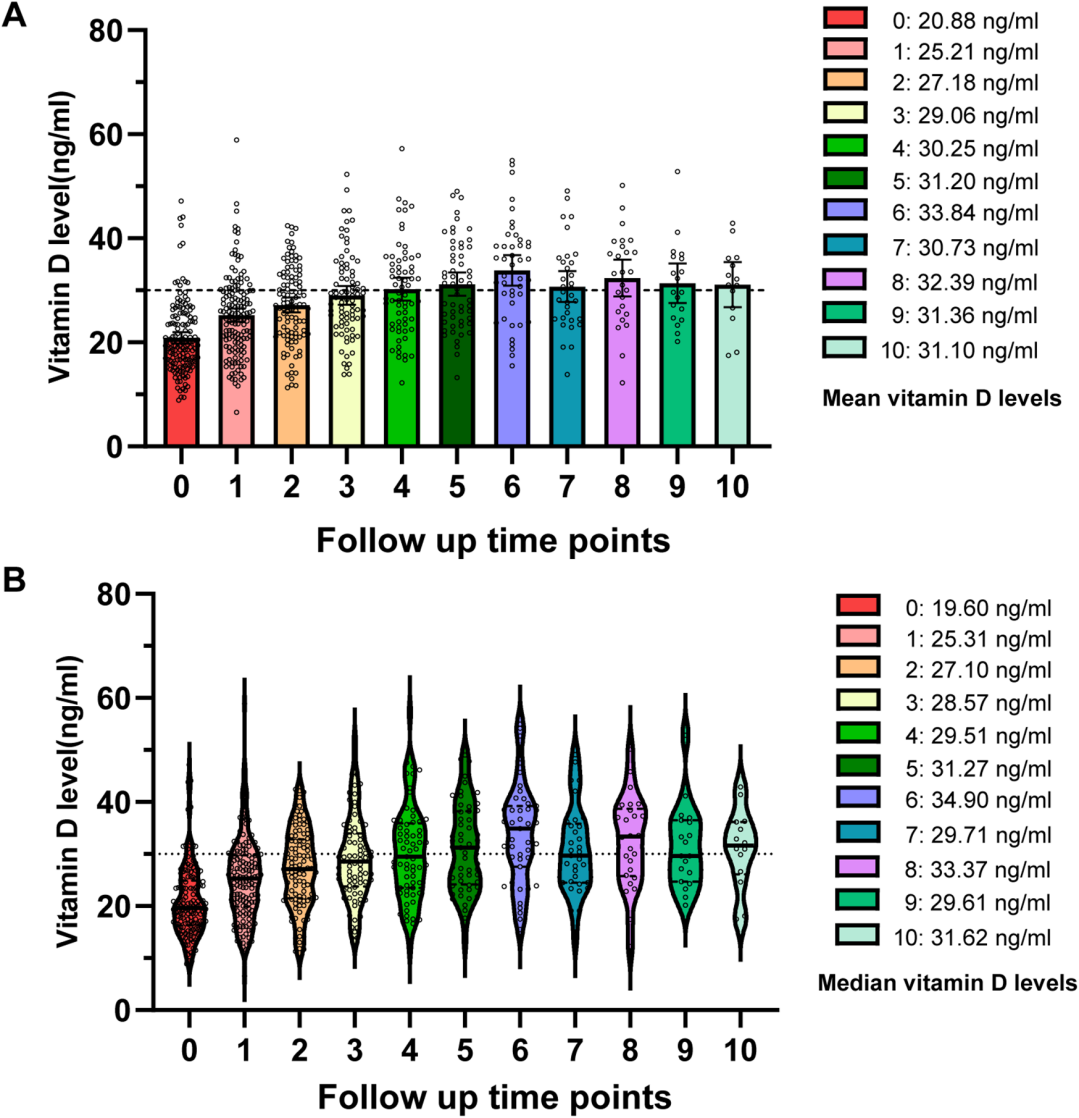
Follow-up frequency and changes in vitamin D levels among girls receiving vitamin D supplementation. **(A)** Scatter plot depicting the mean vitamin D levels at each follow-up point. **(B)** Violin plot illustrating the distribution and median vitamin D levels at each follow-up point.

In order to explore the status of vitamin D supplementation, we performed a follow-up study on 279 girls. Among them, 151 cases continued follow-up and received vitamin D supplementation, and 128 cases loss of follow-up. The 151 cases were classified into three groups based on their baseline vitamin D status: sufficient, insufficient, and deficient (Table 4). We compared their vitamin D levels at the initial visit with those at the first follow-up. It was found that the average vitamin D level of these 151 girls increased from 20.88 ± 6.92 ng/ml to 25.21 ± 7.85 ng/ml after first follow-up (*P* < 0.001). Interestingly, we further investigated the subsequent 10 follow-up records of these 151 girls and found that the mean vitamin D level reached a sufficient level by the fourth follow-up, while the median level exceeded the sufficient threshold by the fifth follow-up (Figure 4). We also analyzed the follow-up data for each subgroup and observed a significant increase in vitamin D levels from baseline to the first follow-up in the early puberty and menarche groups (*P* < 0.001). However, while no significant changes in vitamin D levels were observed in the obesity, short stature, and control groups before and after follow-up (*P* = 0.48, 0.11, and 0.17, respectively), a trend of increased vitamin D levels was clearly observed following supplementation during the follow-up period (Supplementary Figure 1C).

**Table 4:** The first follow-up of vitamin D supplementation.

Group	*N*	Baseline vitamin D (ng/ml, mean ± SD)	First follow-up vitamin D (ng/ml, mean ± SD)	*p*-value	Adherence rate (%)
Sufficient	12	37.07 ± 6.13	36.9 ± 3.44	0.96	81.58
Insufficient	60	24.12 ± 2.85	25.36 ± 2.71	0.2	41.2
Deficient	79	15.96 ± 2.78	23.32 ± 2.91	<0.001	36.04
Total	151	20.88 ± 6.92	25.21 ± 7.85	<0.001	41.11

Baseline vitamin D refers to the vitamin D level of the girls at the time of their inclusion in the study upon their initial visit.

## 4 Discussion

Central precocious puberty is caused by premature activation of the hypothalamic-pituitary-gonadal axis, characterized by the appearance of secondary sexual characteristics in girls before age 8 years (14). Precocious puberty is associated with dietary habits, environmental factors, genetics, and other factors (15–19). In this study, there was a significant difference in vitamin D levels between the early puberty group and the control group, with the deficiency rate reaching 52.07%. This suggests a certain correlation between the occurrence of precocious puberty and the decrease in vitamin D levels. One study have observed that vitamin D levels in children with precocious puberty are significantly reduced, which is consistent with the results of our study (20–23). However, other studies have found no correlation between precocious puberty and vitamin D levels. These difference results from ours could be attributed to regional and population differences (24, 25). A meta-analysis involved 3,016 children with precocious puberty and 8,296 healthy children as the control group, it was shown that children with vitamin D deficiency were more likely to develop precocious puberty (26). The mechanism linking vitamin D deficiency to earlier Gn release remains not fully understood. Studies have shown vitamin D receptors (VDRs) are expressed in the pituitary gland (27). While the presence of VDRs in the pituitary gland suggests a potential role, it does not directly explain the interaction between vitamin D and gonadotropin-releasing hormone (GnRH) pulse generation. Recent studies have shown a negative correlation between VDR gene expression and Gn (28). This suggests that higher VDR activity might be associated with lower Gn release. However, since Gn is generated by GnRH pulses, VDR activity may potentially inhibit GnRH pulse generation through some mechanism. However, current research has not yet confirmed the relationship between VDR gene expression and vitamin D levels. All of the above are potentially pointing to a regulatory role for vitamin D in the hypothalamic-pituitary-gonadal (HPG) axis.

Menarche is a normal physiological phenomenon during female adolescence. With the development of society and the improvement of living standards, the age of menarche in girls is becoming younger (29). The age of menarche has been reported to have associations with some factors such as obesity and genetics (30, 31). The analysis results of this study show a statistical difference in vitamin D levels between the control group and the menarche group. Previous studies have conducted correlation analyses between the age of menarche and vitamin D levels using a research method involving a 30-month follow-up of 242 girls (32). However, in our study, the vitamin D levels in the menarche group were measured when the girls were during the menarche stage, which could directly reflect the physiological status of menarche. Since the onset of menarche marks the full initiation of puberty, and the initiation of puberty requires a certain level of body mass index (BMI), the BMI of individuals who have experienced menarche is significantly higher compared to the control group, and the rate of vitamin deficiency is also extremely high, reaching 73.91%.

Obesity is highly prevalent during the developmental period of children. Obesity can have adverse psychological effects such as low self-esteem, poor academic performance, and depression (33). Furthermore, obesity can have implications for children's physiological health, making them more susceptible to diseases such as diabetes, lipid disorders, cancer, autoimmune diseases, and early-onset polycystic ovary syndrome compared to healthy children (34). The correlation between obesity and vitamin D has also been increasingly validated by research. Studies by Fiamenghi, V.I. et al. and Kasvis, P. et al. have shown that the risk of vitamin D deficiency is higher in children and adolescents with obesity (35–37). Their findings are consistent with the conclusions of this study. while there is a trend toward lower vitamin D levels with increasing age and other physiological indicators, most of these affect is not strong enough to be conclusively determined as significant in this analysis. Further studies with larger sample sizes may be needed to clarify this relationship.

Vitamin D deficiency impairs calcium absorption and disrupts bone mineralization. In response, the body may increase ALP activity in an attempt to promote bone mineralization (38). Consequently, elevated ALP levels are often observed as a compensatory marker of impaired bone health in the context of vitamin D deficiency. In this study, vitamin D deficiency was notably prevalent in the precocious puberty, menarche, and obesity groups, showing a strong correlation with the groups exhibiting elevated ALP levels.

Research on vitamin D in children primarily focuses on investigating the association between vitamin D levels and various health conditions, including its potential relationship between precocious puberty (21). However, the interrelationships between various hormones and vitamin D in different diseases remain to be further explored. In order to investigate the potential correlation between vitamin D and Gn, we conducted a correlation analysis of various indicators in 279 girls. The results showed a significant negative correlation between vitamin D levels and Gn, which was also confirmed in the early puberty group. Within this group, vitamin D was found to be negatively correlated with BMI, Tanner stage, LH and bone age, while showing a significantly positively correlated with blood calcium. This may be attributed to the early activation of the hypothalamic-pituitary-gonadal axis, which accelerates development and growth in girls, leading to the rapid depletion of vitamin D. In the obesity group, vitamin D is significantly negatively correlated with BMI. This phenomenon may be attributed to the fat-soluble nature of vitamin D, which can be stored in adipose tissue and hinder the utilization of free vitamin D, thereby causing vitamin D deficiency in obese children (39).

In this study, all groups exhibited a relatively high deficiency rate of vitamin D. There were significant differences in the deficiency rates of vitamin D between the precocious puberty, menarche, and obesity groups compared to the healthy control group. However, there was no significant difference in the deficiency rate of vitamin D between the short stature group and the control group. This observation may be explained by a hypothesized lower demand for vitamin D due to reduced growth velocity in individuals with short stature. In the study by Lee, H.S. et al., the vitamin D levels in the early puberty group and the control group were 17.1 ± 4.5 ng/ml and 21.2 ± 5.0 ng/ml (*P* < 0.05), indicating vitamin D deficiency in both groups, which is consistent with this study (21). Interestingly, even in the control group, insufficient vitamin D provided a certain indication for supplementing vitamin D in adolescents.

Statistical analysis revealed no significant difference in vitamin D levels in girls between the pre-pandemic and post-pandemic groups in this study. However, Research by Martineau, A.R. et al. indicated an increase in BMI and deficiency rates of vitamin D in children after novel coronavirus epidemic due to reasons such as containment measures (40). It may be related to children's reduced outdoor activities and decreased vitamin D synthesis during the lockdown. However, there is no significant difference in vitamin D levels between the post-pandemic and pre-pandemic periods in girls. This may be attributed to the fact that the girls included in this study were all school-aged children, with a predominance of indoor learning and limited outdoor activities.

Our results suggest that continuous vitamin D supplementation effectively elevated the average serum vitamin D concentration above 30 ng/ml by the fourth follow-up. This outcome underscores the critical importance of timely vitamin D supplementation for girls with developmental variations.

There are several limitations in our study. The sample size needs to be expanded, more vitamin D-related indicators should be included, and further investigation into the underlying mechanisms is required. We plan to address these limitations in future studies on the mechanisms linking vitamin D deficiency and abnormal growth in girls, with the aim of improving and refining our research.

## 5 Conclusion

Our study systematically reviewed the prevalence of vitamin D deficiency among girls with pubertal development variations. The results indicated a common presence of vitamin D deficiency among girls experiencing early puberty, early menarche, and obesity, underscoring the critical importance of timely vitamin D supplementation in this population. Furthermore, this study conducted a preliminary investigation into the association between vitamin D levels and hormonal profiles in these girls, providing a potential foundation for future research on the underlying interaction mechanisms in girls with abnormal pubertal development.

## Data Availability

The original contributions presented in the study are included in the article/Supplementary Material, further inquiries can be directed to the corresponding author.

## Supplementary material

Supplementary Figure S1 **(A)** Distribution of alkaline phosphatase levels across the five groups. **(B)** Differences in vitamin D levels between the pre-pandemic and post-pandemic groups. **(C)** Comparison of vitamin D levels at baseline and first follow-up across five groups. Statistical analysis was conducted using a non-parametric *t*-test. Significance levels are indicated as follows: **P* < 0.05, ***P* < 0.01, ****P* < 0.001.
