# Supplementary figures and images for: Investigation of vitamin D deficiency in girls with growth and development variations—a single center study
Source: Front Pediatr. 2025 Jan 27;13:1518548. doi: 10.3389/fped.2025.1518548 (PMC11808006; doi:10.3389/fped.2025.1518548)

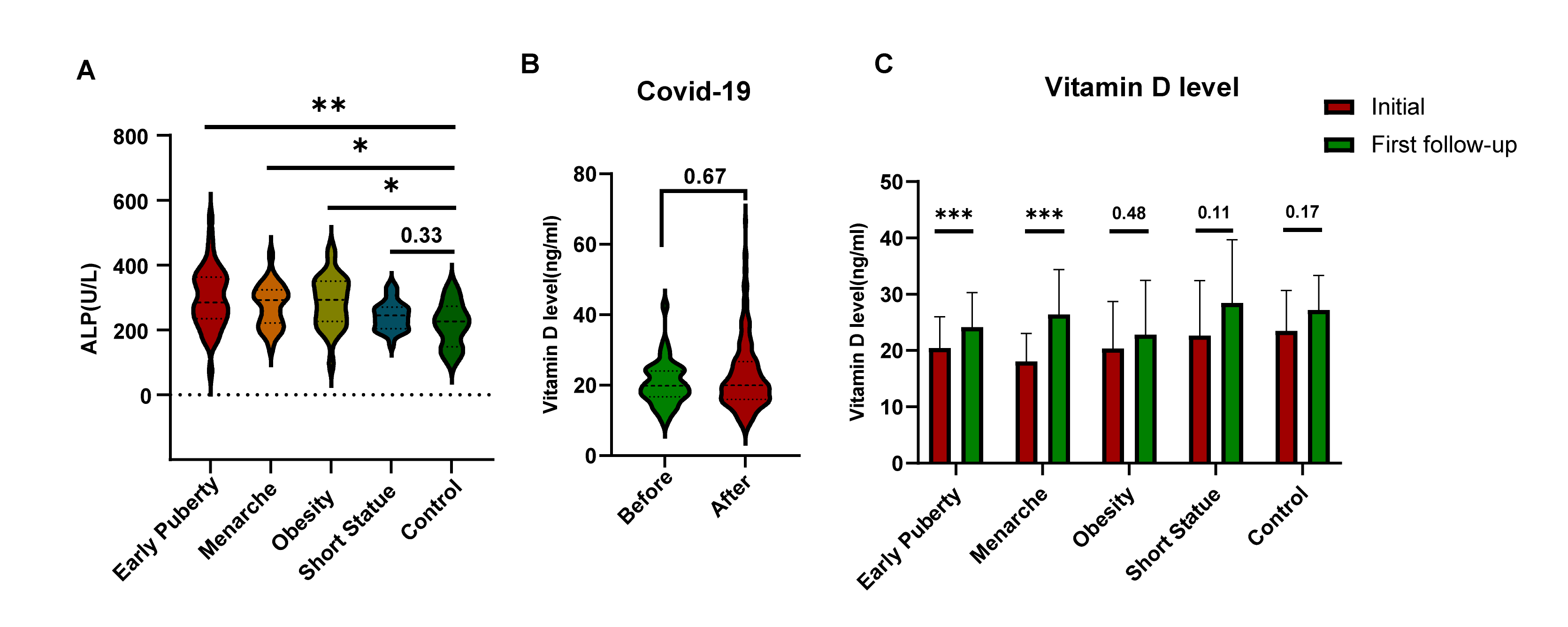

Supplement: Supplementary Figure S1 — (A) Distribution of alkaline phosphatase levels across the five groups. (B) Differences in vitamin D levels between the pre-pandemic and post-pandemic groups. (C) Comparison of vitamin D levels at baseline and first follow-up across five groups. Statistical analysis was conducted using a non-parametric t-test. Significance levels are indicated as follows: *P < 0.05, **P < 0.01, ***P < 0.001. [file Image1.tif]
